# Factors associated with frailty in primary care: a prospective cohort study

**DOI:** 10.1186/s12877-016-0263-9

**Published:** 2016-04-28

**Authors:** Ana Diez-Ruiz, Antonio Bueno-Errandonea, Jazmina Nuñez-Barrio, Inmaculada Sanchez-Martín, Kalliopi Vrotsou, Itziar Vergara

**Affiliations:** Centro de Salud Errenteria-Beraun, OSI Donostialdea, Osakidetza, Errentería, Gipuzkoa Spain; Unidad de Investigación AP-OSIs de Gipuzkoa, Osakidetza, Instituto Investigación Sanitaria Biodonostia, REDISSEC, Paseo Dr. Begiristain s/n, San Sebastian-Donostia, 20014 Spain

**Keywords:** Frailty, Identification, Primary care

## Abstract

**Background:**

Frailty can be defined as a progressive loss of reserve and adaptive capacity associated with an overall deterioration in health that can result in disability, loss of independence, hospitalisation, extensive use of healthcare resources, admission to long-term care and death. Nevertheless, despite widespread use of the term, there is no agreement on the definition of frailty or an instrument to identify it in a straightforward way. The purpose of the current study was to explore which factors are associated with frailty-related adverse outcomes in elderly individuals and to propose a suitable tool for identifying such individuals, particularly in primary care settings.

**Methods:**

A prospective open cohort study of community dwelling, independent individuals aged 75 or over, followed up for 2 years. The study was entirely conducted in a primary care setting. Study variables included independence status measured by Barthel’s Index and the Lawton Instrumental Activities of Daily Living Scale, functional performance, assessed by Timed Up and Go (TUG) and Gait Speed (GS) tests and levels of polipharmacy, comorbidity and social support. Outcome variables were specific frailty-related adverse events, namely, loss of independence and death.

**Results:**

Overall, 215 community-dwelling independent individuals initiated the study. Of these, 46 were lost to follow-up and 50 had frailty-related adverse events during the follow-up period. Individuals with adverse events during the study had poorer functional status at baseline. The multivariate model that best explained the occurrence of these events included the variables of age, presence of polipharmacy and the TUG time. The AUC (Area under the curve) of this model was 0.822.

**Conclusions:**

Given the simplicity of assessing the three derived factors and their combined discriminant power, the proposed model may be considered a suitable tool for identifying frail patients, i.e., people more likely to lose their independence or die within a relatively short time interval.

## Background

Frailty can be defined as a progressive loss of reserve and adaptive capacity associated with an overall deterioration in health that can result in disability, loss of independence, hospitalisation, extensive use of healthcare resources, admission to long-term care and death [1–3]. Nevertheless, despite the widespread use of the term, there is no agreement on the definition of frailty [4, 5] or on an instrument to identify it [6] in a straightforward way, particularly in primary care.

Three approaches for the identification of frail individuals have been described in the literature [7]: the rules-based, the cumulative and the clinical judgment. Rules-based approaches are derived from multiple regression models and are based on the presence of a number of symptoms. The phenotypic approach would be included in this group [1]. Cumulative-based approaches are based on the consideration and addition of the number of impairments presented by a single person [8]. Finally, clinical judgment based approaches rely on the professional interpretation of the clinical record and physical examinations [7].

A frequently considered aspect in the aforementioned approaches is functional performance. Several instruments have been proposed to assess various aspects of functional performance such as motor strength, mobility and balance. Those most commonly used being the Timed Up and Go (TUG) [9, 10] and Gait Speed (GS) tests [11, 12]. Such tests have demonstrated their usefulness in terms of monitoring the health status, functional capacity and risk of falls of elderly people [11, 13–15].

Early identification of frail individuals in primary care could have a double impact. On the one hand, it may help improve the health status of identified individuals, since it would be possible to address their needs in a comprehensive manner, delaying or preventing the natural progression towards dependence [16]. On the other hand, it may lead to reductions in resources use [2].

The objective of this study was to assess which factors are associated with frailty-related adverse outcomes in elderly individuals and on the basis of these results and from a rules-based perspective, to propose a suitable instrument for identifying these patients in primary care.

## Methods

### Study population and recruitment

An open cohort of community-dwelling individuals, aged 75 years or more and independent (Barthel’s index ≥90) at the time of inclusion. Patients of advanced age were selected in order to assure a rate of occurrence of the defined adverse outcomes, adequate to the project design and span [17, 18]. Recruitment took place in the primary care centres of two municipalities in Gipuzkoa (Spain) and subjects were followed up for 2 years. The participating health centres are situated in an urban area close to the provincial capital (San Sebastian) and their population characteristics are similar to the Basque Country region in terms of age, sex and deprivation index [19]. These centres provide primary care services to a total adult population of around 30,000 individuals. The study was carried out between July 2010 and December 2013.

Eligible individuals were selected randomly from administrative databases in order to obtain a representative sample of the reference population in terms of age and sex. After being informed about the study by their corresponding primary care doctors they were invited to a meeting where detailed information about the research project was presented and informed consent was provided. The study was approved by the Clinical research ethics committee of the Gipuzkoa health region (ref.: 05/2010).

Individuals were excluded from the study if they were institutionalised, planned to move within the following 2 years or were included in a home care programme for chronic health problems. Patients with impaired cognitive function, defined as 5 or more errors on the Pfeiffer’s Short Portable Mental Status Questionnaire [20, 21], and those who were dependant, defined as a score <90 in the Barthel’s index [22] were also excluded. All participants provided informed consent.

### Study variables

As main outcome variables were considered the events of death and loss of independence; the latter defined as loss of ≥ of 10 % of the baseline Barthel score during the study follow-up period, considering that this reduction on Barthel score imply the loss of independence in preforming a basic daily living activity [23–27].

Interviews were performed on an individual basis and information collected at baseline included among others: age, sex, level of education, living arrangements, and social risk measured using the Gijon Scale [28]. To assess participants’ functional status, the Lawton Instrumental Activities of Daily Living (IADL) Scale [23, 29] and two physical functional performance tests (TUG and GS) [9, 11] were used. To assess overall health status, information was requested regarding loss in body weight, level of physical exercise, sensory deficits including hearing impairment, and self-perceived health using the following question: “How would you rate your health?”

Additionally, health records were reviewed to detect any falls and hospital admissions in the previous year; to confirm the presence/absence of polipharmacy, defined as the simultaneous prescription of 4 or more drugs continuously for 3 or more months [30]; and/or comorbidity, defined as three or more of the following conditions: stroke with sequelae, myocardial infarction or recently diagnosed heart failure, Parkinson’s disease, chronic obstructive pulmonary disease, musculoskeletal disorders resulting in pain or functional impairment, diabetes mellitus treated orally or with insulin, neurological gait disorders, unresolved urinary complaints, and mental illness under treatment with psychoactive medication [31]. The interviews and review of health records were carried out by two nurses trained for the purpose.

### Follow-up

Every six months from the date of recruitment and over the 2 years of follow-up, patients were appointed for an additional assessment. These assessments included all the aforementioned functional and daily activity measures (Barthel’s index, Lawton’s scale, TUG and GS tests) and information about weight, physical exercise, sensory deficits and self-perceived health. The corresponding nurse was contacting patients not attending a follow-up assessment visit. Those stating that they lost interest in the study were considered drop-outs. Patients who lost independence and had to be admitted to an older people’s home were registered as dependent. If the family took care of the dependent individuals, Barthel values were also collected by interviewing the person responsible to provide for them. Death events were verified by medical records. Patients who could not be located and did not come back for the rest of the assessments were considered drop-outs. Patients, who returned after missing certain assessments, were kept in the final sample and were assigned in the corresponding outcome group. No missing values imputation was performed for the needs of this study.

### Sample size

Loss of independence and mortality rates in subjects over 75-years of age were reviewed in previous studies in our setting (non-published data). Obtained estimates were equal to 18 and 5 % respectively. Assuming a dropout rate of 10 % during the follow-up period, we calculated that with *n* = 200 participants we could expect around 40 individuals to lose their independence by the end of the study. Given the binary nature of the considered outcome (deterioration Yes/No), applying the rule of thumb of 10 events per variable this sample size would enable us to construct binary logistic regression models including simultaneously 4 to 5 predictive factors, should results indicate that many factors to be relevant.

### Statistical analysis

The unit of analysis was the patient. Descriptive statistics were used to analyse dependent and independent variables at baseline and each follow-up assessment. Frequencies and percentages were calculated for categorical data and means and standard deviations (SD) or medians and interquartile ranges (Q1, Q3) for continuous data, depending on their distribution. Categorical variables were compared with chi-squared or Fisher’s exact tests while Student’s *t*-test or the non-parametric Wilcoxon were implemented for normally and non-normally distributed continuous variables.

In order to assess whether missing data were random or were associated with specific patient characteristics, socio-demographic and clinical characteristics variables were compared between lost to follow-up individuals and those who completed the follow-up. Finally, univariate and multivariate binary logistic regression models were constructed to assess the relationship between each of the independent variables and the occurrence of the frailty-related adverse events under study. All variables with significance levels *p* ≤ 0.10 in the univariate analysis were considered for the construction of the multivariate model. At this stage both backward and forward stepwise models were tested. In the backward procedure all relevant variables entered in the same model. Non-significant variables were eliminated one-by-one, based on their p-values (eliminating the less significant first), until no *p*-value >0.05 were left in the model. In the forward procedure, the most significant variable entered in the model, followed by the next more significant etc., eliminating those with *p*-value > 0.05 [32].

In a secondary stage sensitivity analyses were performed. Further multivariate models were fitted, making different assumptions about the drop-out patients. In particular it was assumed that: a) all lost to follow-up had a negative outcome; b) all lost to follow up had a positive outcome; c) those not located had a negative outcome and finally d) those not located had a positive outcome.

The diagnostic performance and goodness-of-fit statistics were studied for all constructed models. The results of the final model are presented in terms of odds ratios (OR) with corresponding 95 % confidence intervals (CI). The area under the curve (AUC), Hosmer-Lemeshow statistic and R-square values are also reported. Results were considered statistically significant when *p* <0.05. All analyses were performed with the SAS 9.3 software.

## Results

**Fig. 1:**
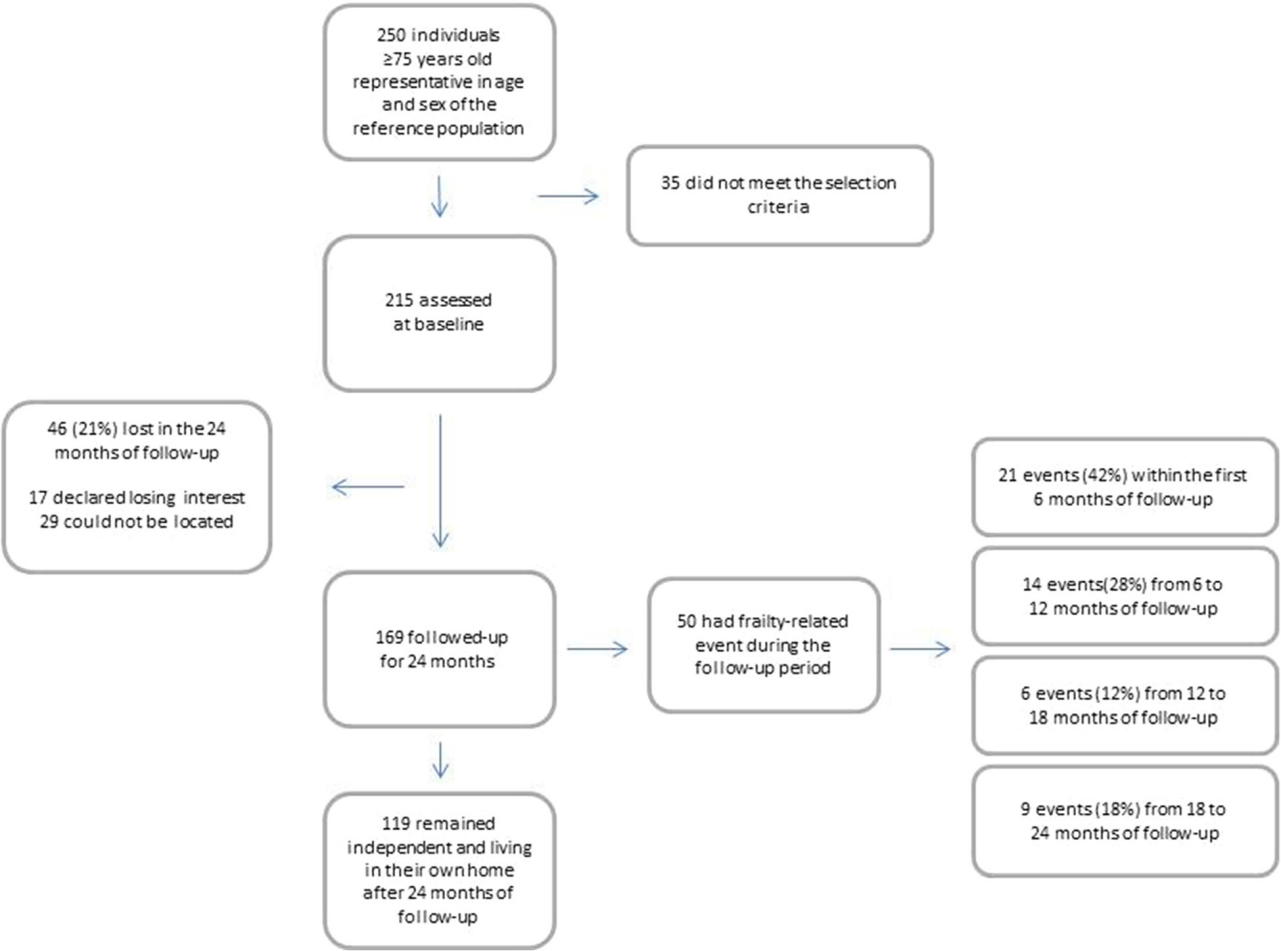
Flow chart of patient recruitment and follow-up

A total of *n* = 215 individuals were initially included in the study and of those *n* = 169 completed the proposed follow-up. No significant differences were found in terms of socio-demographic and clinical characteristics between lost to follow-up subjects and those who completed the study. At the end of the follow-up period, *n* = 119 subjects remained independent, while the other *n* = 50 participants (24 %) had a frailty-related adverse outcome: death (*n* = 8) or loss of independence (*n* = 42) (Fig. 1). The rates of independence loss in the first and second years were 8.3 and 2.6 %, respectively.

At baseline, participants had a mean age of 79.4 (SD: 4.1) years and 63 % were women. A high proportion of subjects presented comorbidity (72 %) and polipharmacy (61 %). Comparing the baseline status of those who eventually developed a frailty-related adverse outcome and those who did not, we observed the following. The adverse outcome group was on average 3 years older (*p* < 0.001), had a lower level of physical activity (*p* = 0.001) and was more likely to present polipharmacy (*p* = 0.001) or comorbidity (*p* = 0.032). In addition, it presented more hospital admissions in the previous year (*p* = 0.013) and declared a poorer self-perceived health status. No significant differences were found in the variables of sex, body mass index, visual or auditory deficits and accidental falls in the previous year (Table 1).

**Table 1:** Baseline data of the entire sample and comparison between the two groups of adverse events

		Adverse event		
Baseline variables	Total (*N* = 215)	Yes (*n* = 50)	No (*n* = 119)	*p*-value
Age; mean (SD)	79.4 (4.1)	81.7 (4.6)	78.4 (3.5)	<0.001
Sex: Female	136 (63)	32 (64)	76 (64)	0.987
Able to read and write	205 (95)	47 (94)	114 (96)	0.695
Living with spouse of family member	162 (75)	35 (70)	88 (74)	0.599
Weight loss in the last year^b^	18 (8)	6 (12)	5 (4)	0.085
Low level of physical activity	22 (10)	11 (22)	6 (5)	0.001
Polipharmacy	131 (61)	41 (82)	65 (55)	0.001
Fall in the previous year	52 (24)	12 (24)	30 (25)	0.868
Hospital admission in the previous year	36 (17)	13 (26)	13 (11)	0.013
Self-perceived health				
Good/very good	136 (63)	26 (52)	91 (77)	0.003
Fair	73 (34)	23 (46)	26 (22)	
Poor/very poor	7 (3)	1 (2)	2 (1)	
Social risk (Gijon); median (Q1, Q3)	10 (9, 12)	10 (8, 12)	10 (9, 12)	0.442
Comorbidity: Yes	153 (72)	35 (70)	62 (52)	0.032
Presence of health problems ^a^				
Body mass index >30 kg/m^2^	63 (29)	18 (36)	33 (28)	0.285
Musculoskeletal disorders	52 (24)	17 (34)	21 (18)	0.020
Diabetes under treatment	37 (17)	8 (16)	23 (19)	0.610
Chronic obstructive pulmonary disease^b^	12 (6)	6 (12)	1 (1)	0.003
Visual deficit ^b^	8 (4)	3 (6)	2 (1)	0.154
Auditory deficit ^b^	6 (3)	1 (2)	4 (4)	1.000

Adverse event: death or loss of independence defined as ≥10 % drop in Barthel’s score compared to baseline, during the follow-up. Data are expressed as frequencies (percentages), unless otherwise stated. For dichotomous variables one of the two categories are presented.

^a^Presented health problems are not exclusive; a patient can suffer by more than one. P values in the last column refer to comparisons between the groups with and without adverse events (Yes vs. No). Age was compared with Student’s *t* test

^b^these variables were compared with Fisher’s exact test, the Chi-square test was implemented for the rest of the variables

As far as functional status is concerned, participants who developed frailty-related adverse outcomes, despite being independent at baseline, had lower levels of functioning in terms of basic activities of daily living (Barthel’s index) and IADL (Lawton’s score). Significant differences were also observed in the functional performance tests of TUG and GS at baseline. Participants with frailty-related adverse outcomes performed worse in both (*p* < 0.001) (Table 2). No socio-demographic differences were observed between those finishing the study and drop-out subjects. The latter were half a year older than the rest (mean drop-out age 79.9 (SD: 4.0), *p* = 0.398); 39 % of them were men, compared to 36 % of those completing the study (*p* = 0.705). Both groups had similar baseline Barthel values (*p* = 0.901) and self-perceived health (*p* = 0.218).

**Table 2:** Baseline data on functioning and comparison between groups with and without adverse frailty-related outcomes

	Adverse event		
Functional tests	Yes (*n* = 50)	No (*n* = 119)	*p*-value
Barthel’s index; *n* (%)			
90 points	16 (32)	7 (6)	<0.001
95–100 points	34 (68)	112 (94)	
Lawton IADL; median (Q1, Q3)	6 (4, 8)	8 (5, 8)	<0.001
Timed Up and Go time, s; median (Q1, Q3)	15 (13, 22)	12.5 (11, 14)	<0.001
Gait Speed, m/s.; mean (SD)	0.8 (0.2)	1.1 (0.2)	<0.001

Categorical variables were compared with the chi-squared test; means and medians were compared using the Student’s t and Wilcoxon tests, respectively

*Adverse event* death or loss of independence defined as ≥10 % drop in Barthel’s score compared to baseline, during the follow-up, *IADL* Instrumental activities of daily living. The abbreviations: *s* and *m/s* indicate seconds and meters per second respectively. *Q1, Q3* interquartile range from the first to the third quartile, *SD* standard deviation

Except for Barthel and Lawton scales, all other variables with *p*-values ≤0.10 presented in Tables 1 and 2 were implemented in the construction of the multivariate logistic regression model. It’s worth mentioning that due to their high correlation (*r* = −0.821), GS and TUG could not be both considered in the same model and the TUG was selected given its capacities [33]. Furthermore, the presence of comorbidity and having specific health problems (e.g., musculoskeletal disorders) were not significant in any of the models that included the polipharmacy variable, possibly due to the strong association between these factors. Both the forward and backward stepwise regressions resulted in the same multivariate model.

**Fig. 2:**
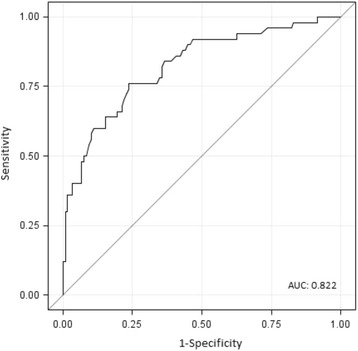
ROC of the proposed model for identifying frailty in primary care. Receiver operating characteristic curve for the final model to predict frailty-related outcomes, based on age, Timed Up and Go time and polypharmacy status. The curve represents the relationship between sensitivity and 1-specificity for all potential cut-off points of the diagnostic test under study. The area under the curve (AUC), a measure of the discriminatory power of the test, is 0.822. The cut-off point that maximises sensitivity and specificity (i.e., 76 %) is 0.288

The multivariate logistic regression model that best fitted the data included age (OR: 1.14; 95 % CI: 1.03, 1.25), polipharmacy status (OR: 2.74; 95 % CI: 1.06, 7.06) and TUG time (in seconds) (OR: 1.28, 95 % CI: 1.14, 1.44) (Table 3). Our results suggest that these factors have good discriminant validity, with an area under the curve (AUC) of 0.822 (Fig. 2), adjusted R-squared of 0.384 and Hosmer Lemeshow *p* = 0.721 (Table 3).

**Table 3:** Multivariate logistic regression model for the onset of adverse events related to frailty

Variable	Odds ratio	95 % CI	*p*-value
Age, years	1.14	1.03, 1.25	0.012
Timed Up and Go time, s	1.28	1.14, 1.44	<0.001
Polipharmacy			
No	Ref	–	–
Yes	2.74	1.06, 7.06	0.037
Goodness-of-fit statistics			
Area under the curve: 0.822			
R squared / adjusted R squared: 0.270/ 0.384			
Hosmer-Lemeshow: *p* = 0.721			

The probability of suffering a frailty-related adverse event during the follow-up period was modelled. Adverse event: death or loss of independence defined as ≥10 % drop in Barthel’s score compared to baseline, during the follow-up, 95 % CI: 95 % confidence interval. Polypharmacy: long-term prescription of ≥ 4 drugs. The model is based on *n* = 50 adverse events and *n* = 118 positive events due to 1 missing value in TUG test

The additional sensitivity analyses gave the following results. When all lost to follow-up (*n* = 46) were assumed to be finally dependent, the derived model included the variables of age, TUG and at least one hospital admission in the last year. When only those not located (*n* = 29) were considered dependent, the model included age and TUG. When all lost to follow-up (*n* = 46) or only those not located (*n* = 29) were assumed to be independent, the final models included the variables of age, TUG and polipharmacy. The AUCs of all four models oscillated between 0.733 and 0.795.

## Discussion

Proper identification of frail individuals in primary care represents an unresolved challenge. The current study, leaving aside discussions on the definition of frailty and its components, addresses this issue from a pragmatic point of view. Subjects able to perform basic daily living activities (BADL), so independent, are considered frail when they lose independence or die in a maximum period of two years. With this approach, the prevalence of frailty in our sample would be 24 %, higher than expected in our setting, but in agreement with other published studies [4, 34].

Our study shows that it is possible to identify clear differences between groups of community-dwelling elderly individuals that do and do not lose their independence within 2 years. The differences are significant in terms of age, level of physical activity, health status and functional capacity. The current results are in agreement with the literature on this topic, indicating association between frailty with age, comorbidity, self-perceived health and functional status [35]. No differences in terms of socioeconomic status, or family and social network were found in our sample.

Considering the prospective nature of the methodology used in this study and the rules-based approach proposed for the construction of operational definitions of frailty, we found that the factors associated with frailty-related adverse outcomes could be expressed in a single model including age, polipharmacy status and TUG time. We should underline that when constructing the model polipharmacy was selected over other comorbidity-related variables, as it is easier to assess than other comorbidity indices. As stated in the results section, none of these other variables was statistically significant when considered simultaneously with polipharmacy, most likely due to the existing relations between them. The sensitivity analyses performed seems to support the presented model. Even though hospital admission turned out to be significant, when all drop-outs were considered dependent, it is a fact that hospital admission and polipharmacy are not independent, rather highly related variables. Recent hospital admission may be indicative of a health problem which can end up in a negative outcome and has been often described as associated to frailty [36]. A high association between the two was also found in our in our sample (*p* = 0.002).

To date, a variety of approaches have been suggested for identifying frail individuals. One well-known approach uses the frailty phenotype proposed by Fried in 2001 [1], based on the presence of at least three of the following deficits: slow gait speed, weak grip strength, low level of physical activity, exhaustion and unintentional weight loss. Despite the widespread use of this phenotype in research, this tool has not been widely adopted in clinical practice, possibly due to the lack of population studies establishing diagnostic cut-offs for some of the criteria [3]. Other ways of identifying frail individuals involve frailty indices, based on cumulative approaches [37]. Indices published in recent years include the Survey of Health, Ageing and Retirement in Europe (SHARE) Frailty Instrument [38, 39], and the Program of Research to Integrate Services for the Maintenance of Autonomy (PRISMA) questionnaire [40]. These indices assess the existence of deficits in areas such as strength, balance, nutrition, resistance, mobility, physical activity and cognitive ability, but none of them have been taken up in primary care settings. Also, a third approach based on the clinical judgment it’s being explored. In this group, the Gérontopôle Frailty Screening Tool [41] and the EASY-Care TOS [42, 43] can be considered. The latter, with a specific focus on primary care settings, provides a robust and easy to perform two step tool for the identification of frail patients in primary care practices. It is interesting to note the parallelisms with this article especially regarding aim (primary care oriented) and methodology (longitudinal, adverse outcome based). Nevertheless, the main difference is the implemented frailty identification approach. Our tool is a rules-based one while EASY-Care TOs is based on clinical judgment. Additionally, our proposal overcomes one of the main limitations of the clinical judgment based tools, which is that they rely on a strong and long term patient –care professional relationship, whereas the proposed tool is based on three objective and easy to measure variables, not requiring previous knowledge of the patient’s clinical history. Hence, given the characteristics and simplicity of our model, we believe that once validated in a different and broader population, it could be considered as a screening test for identifying frail individuals in primary care.

The main limitation of our study is related to its cohort design and associated losses to follow-up. To minimise this bias, we thoroughly informed patients about the goals of the research, arranged informative meetings with them and carried out a campaign to raise awareness of the study in the participating health centres. There were more losses during the follow-up period than expected, but figures were similar to those of other studies and are considered acceptable [44]; further, we reached the number of frailty-related adverse outcomes expected, which has enabled us to achieve the desired robustness in the analysis. Another notable limitation is the fact that repeating performance tests may lead to a certain degree of training. In any case, this limitation would underestimate the onset of functional deterioration and hence would not affect the validity of the results [45]. To minimise bias from inter-observer differences, we standardised the criteria for the administration of the various different scales and tests and trained the nurses in charge of data collection.

The main strength of this study is the fact that it was conducted entirely in a primary care setting. Many studies in this field are conducted in geriatric settings, where population characteristics and patient needs are very different. A recently initiated research project (reference PI14/01905) will allow us to further validate the ability of the proposed model in identifying frail individuals in a larger sample of independent community dwelling elders aged 70 or more.

## Conclusions

It is possible to identify frail individuals considering their age, polipharmacy status and functional capacity. These three factors can be assessed in a simple and quick way, fact which renders the proposed model suitable to use in primary care.

### Ethics approval and consent to participate

All participants received complete information about the project and written informed consent was provided. The study was approved by the Clinical Research Ethics Committee of the Gipuzkoa Health Region (ref.: 05/2010).

### Consent for publication

Not applicable.

### Availability of data and materials

The database set was available for all authors of the study and will be available for other non-commercial researches on request.

## Abbreviations

AUC: area under the curve
BADL: basic activities of daily living
CI: confidence intervals
GS: gait speed
IADL: Instrumental activities of daily living
OR: odds
PRISMA: Program of research to integrate services for the maintenance of autonomy
SD: standard deviations
SHARE: Survey of health, ageing and retirement in Europe
TUG: Timed up and go
